# Kickstarting the First Coronary Artery Bypass Graft Program in Papua New Guinea—History Made, Yet a Long Journey Ahead

**DOI:** 10.3390/jcm15124763

**Published:** 2026-06-18

**Authors:** Ling Zhu, Kim Chai Chua, Daobo Wang, Daniel Kanasa, Arvin Wesley Karu, Oriana Ng, Noah Tapaua, Yeow Leng Chua

**Affiliations:** 1 Department of Cardiothoracic Surgery, National Heart Centre Singapore, 5 Hospital Drive, Singapore 169609, Singapore; chua.kim.chai@singhealth.com.sg (K.C.C.); daobo.wang@mohh.com.sg (D.W.); chua.yeow.leng@singhealth.com.sg (Y.L.C.); 2 Department of Cardiothoracic Surgery, Port Moresby General Hospital, Boroko 111, National Capital District, Port Moresby 111, Papua New Guinea; dkkanasa@gmail.com (D.K.); ntapaua@yahoo.com (N.T.); 3 Department of Anesthesiology, Port Moresby General Hospital, Boroko 111, National Capital District, Port Moresby 111, Papua New Guinea; trentkaru@yahoo.com; 4 Department of Anesthesiology, Block 2 Level 2 Singapore General Hospital, Singapore 169608, Singapore; oriana.ng@singhealth.com.sg

**Keywords:** CABG, Papua New Guinea, low- and middle-income country, cardiac surgery program

## Abstract

**Background/Objectives**: Papua New Guinea has a population of over 10 million, with its public cardiac surgical service provided by only one tertiary center. Despite the climbing burden of ischemic heart disease, no CABG operation has been performed before 2025 due to limited local surgical capacity. An international collaboration was planned in order to launch a CABG program in the country. **Methods**: Three cases were shortlisted after a multidisciplinary team discussion. A team-based “On-the-job” mentoring strategy was employed to facilitate skill transfer. The operation was carried out in a “twinning” fashion, with each role of the surgical team being taken up by “a pair”—the trainer (visiting team) and the learner (local team). The trainer demonstrated key skills and tips in the first case, and the “pair” switched positions in the following cases to maximize hands-on learning. The last case was performed entirely by the local team. **Results**: Three patients underwent CABG operations in this pilot program. A total of 2.33 grafts/case were performed on average, with no 30-day mortality. There were no major complications except for one patient developing right middle cerebral artery infarct on postoperative day 5. The patient was discharged one month later after achieving functional recovery and was started on anticoagulation therapy. **Conclusions**: International collaborations with strategic planning can play a critical role in starting new cardiac surgical programs in low–middle-income countries, with acceptable surgical outcomes. History has been made with the first-ever CABG operation successfully performed in Papua New Guinea. The journey ahead to sustain local cardiac surgical capacity and to provide safe and accessible cardiac surgical care for the country remains challenging.

## 1. Introduction

Papua New Guinea (PNG) is a lower-middle-income country (LMIC) with a population of over 13 million and approximately 250,000 live births annually. The burden of congenital and acquired heart disease is substantial; however, public cardiac surgical service in the country is currently provided by only one national heart center located in the capital city of Port Moresby. International mission trips over the last 30 years by foreign medical teams have provided lifesaving care, mainly focusing on congenital heart conditions and adult valvular diseases. Despite the exact epidemiology remaining unclear, the burden of cardiovascular disease has been surging in PNG, with pressing clinical needs. The first catheterization laboratory in the country was opened in 2019. No coronary artery bypass graft (CABG) had yet been performed until 2025 due to limited local surgical capacity and impediment by the COVID-19 pandemic. An international collaboration between the local cardiac team from the National Heart Centre of PNG and a visiting team from Singapore was planned in order to start the first CABG program in PNG.

## 2. Materials and Methods

### 2.1. Personnel

The visiting team from Singapore (the “visiting team”) had previously established a long-time partnership with the PNG national heart center located in Port Moresby General Hospital (the “local team”), centering on cardiovascular disease treatment and specialty training since 2013. Past mission trips have focused on valvular surgeries, with satisfactory surgical outcomes [1]. A new multidisciplinary team (the “Team”) was formed this time for the pilot CABG program, comprising cardiac surgeons (including a specialized coronary surgeon), cardiologists, cardiac anesthetists, senior trainees, perfusionists, and operating theater (OT) and intensive care unit (ICU) nurses from both the local team and the visiting team. The visiting team was skeletonized to a minimum size of 8 members to cover the essential perioperative components involved in CABG surgery while allowing for maximal autonomy and practical experience for the local team. Topics centered on the perioperative management of patients undergoing CABG operations were introduced via targeted teaching materials, and teaching sessions were carried out prior to the surgeries.

### 2.2. Patients

A total of ten adult patients aged between 44 and 65 years with ischemic heart disease were referred by physicians from all over the country to the national heart center for consideration for bypass surgery. Their baseline demographic, clinical, coronary angiogram, and echocardiographic data were collected and screened. Four patients were identified as candidates for CABG by the Team after online discussion. Selection criteria were based on the patient’s symptoms, complexity of the coronary artery disease, surgical risk profile, social support, etc. At the start of the program, “straight-forward” cases with satisfactory target vessels and standard risks were preferred. Three patients were shortlisted after physical review at bedside and underwent surgeries in Aug 2025.

### 2.3. Operative Approach

As the local team was already experienced in congenital and valvular operations, a role-based “on-the-job” mentoring strategy was employed to facilitate skill transfer for CABG. Briefing (“Huddling”) was done in the OT before the start of each case, recapturing the patient’s details, operation plan (coronary targets and bypass grafts to be harvested), and specific concerns. The surgical approach was standardized for every case. After median sternotomy, the left internal mammary artery (LIMA) was harvested. The saphenous vein +/− radial artery was harvested simultaneously via the open method. After intravenous heparin, the ascending aorta and right atrium were cannulated to initiate cardiopulmonary bypass (CPB) with moderate hypothermia (30–32 °C). After aortic cross-clamping (AXC), antegrade cold blood cardioplegia was administered down to the aortic root. Distal anastomoses were performed on the arrested heart. After AXC was removed while rewarming, proximal anastomoses were performed under a side-biting aortic clamp. After completion of all anastomoses, the patient was gradually weaned off from CPB, before hemostasis and chest closure.

The first CABG operation was carried out in a “twinning” fashion, with each role of the surgical team (the chief surgeon, first assistant, conduit harvester, anesthetist, perfusionist, and OT and ICU nurses) being taken up by “a pair”—the trainer (visiting team) and the learner (local team). The trainer led the surgery by demonstrating key skills and tips for CABG. Moving onto the second case, the “pair” switched their positions. The learner maximized the hands-on time to actualize what they had learned, while the trainer assisted as needed. The third case was then piloted and performed entirely by the local team—with only proctoring required by the visiting team on the side.

### 2.4. Statistical Analysis

Informed consent for data collection and research was obtained from the patients after approval by the hospital research ethical committee. Comprehensive data collection at baseline such as demographics, medical history and risk factors, procedural data, medications, complications, and discharge outcomes were collected prospectively in the local center database. Statistical analysis was performed with Microsoft Excel (Version 2024). Baseline demographics and clinical characteristics were summarized using descriptive statistics. For continuous variables, data were presented as mean or median and standard deviations or interquartile range. Categorical variables were presented as absolute numbers and percentages.

## 3. Results

### 3.1. Demographic Data

A total of three patients (two male and one female) successfully underwent the CABG operation under this pilot program. The patients’ characteristics and perioperative variables are summarized in Table 1. The mean age at operation was 51.7 ± 7.0 years (range 45–59). Left ventricular ejection fraction was mildly impaired in two cases. All patients had equal functional classes, corresponding to New York Heart Association (NYHA) Class II. The mean EuroSCORE II was 0.89 ± 0.11%.

### 3.2. Operative Outcomes

The average grafts performed were 2.33 vessels/case. The LIMA was used for all three cases, and a radial graft was used for one of the younger patients with a suitable target. The mean CPB time was 92.0 ± 21.0 min, and mean AXC time was 58.3 ± 21.6 min. There was no case that required a second pump run or repeated cross clamping. Two patients required postoperative blood transfusions of packed cells. All the patients were extubated overnight after the surgery. Antiplatelet therapy and other medical therapies were started according to guidelines [2]. The average ICU stay was 1 day, and there was no 30-day mortality. All patients developed postoperative atrial fibrillation (AF) and were treated pharmacologically. The second case developed AF on postoperative day (POD) 2 and developed right middle cerebral artery infarct on POD5, with left-sided weakness. She was discharged 1 month later after achieving functional recovery. She also reverted to sinus rhythm and was continued on anticoagulation therapy.

At the last follow-up at 4 months, there was no delayed mortality. The patients were continued on antiplatelet therapy, and risk factor control for ischemic heart disease was achieved, with functional status improving to NYHA class I. Two patients returned to their original provinces to continue the follow-up with the local physicians. The patient who suffered a major stroke post-surgery returned to her baseline functions.

## 4. Discussion

PNG is an LMIC with over 150 islands in the South Pacific region, with more than 80% of the population still living in rural areas. PNG serves as an important regional hub to provide medical care for not only its own population but also the population of the over 600 offshore islands nearby, ranging from small coral atolls to larger, mountainous, and volcanic land masses, with a catchment area of over 10 million people [3]. PNG is one of the countries with the highest rates of rheumatic heart disease [4] but is also witnessing a rapid rising trend in ischemic heart disease, in parallel with urbanization, which serves as one of the top leading causes of death other than communicable diseases [5]. Moreover, the limited cardiac surgical services in PNG are far from meeting the needs of the high disease burden this country is facing [6].

Cardiac services in PNG started with a small cardiac unit at the Angau Memorial hospital in Lae in the early 1970s with support by an Australian team, but this was closed after PNG gained independence in 1975. The Operation Open Heart program in collaboration with the Australian team started visiting the country in 1993, operating on congenital heart disease [7]. The Singapore team started their visits in 2013, focusing on rheumatic heart disease, with three mission trips completed between 2015 and 2017 and a total of 20 operations performed with satisfactory operative outcomes [1]. The visiting team initially operated at full capacity, comprising senior surgeons, an echocardiologist, an anesthetist, a perfusionist, and OT, ICU, and ward nurses. Besides service delivery, the main aim of the project was to train the local team on capacity building while progressively reducing the scale of the visiting team to allow the local workforce to gain independence. Between 2014 and 2016, one cardiac surgeon, two cardiologists, one cardiac anesthetist, two perfusionists, and six nurses (from the OT, ICU, and ward) from PNG also completed their overseas training in Singapore. In 2019, a new era began with the opening of the PNG National Heart Centre at Port Moresby General Hospital, equipped with a catheterization lab, cardiothoracic ward, and cardiac operating theater, moving towards a full cardiac specialty service in the country. The trajectory came to a sudden halt with the advent of the COVID-19 pandemic. The local team had to rely solely on its own capacity while international visits were put on hold. Many of the patients with severe ischemic heart disease were left untreated and undertreated due to limited access to diagnostic angiograms and percutaneous coronary intervention, and no available CABG service.

Collaboration with the Singapore cardiac team managed to be resumed in 2025. The goals were refurnished to strengthen the existing services such as valve and congenital surgeries and, more importantly, to establish the expertise of the local team in performing CABG operations. In contrast to open-heart operations, a bypass operation is precluded by conduit preparation, and each conduit harvest stands alone as an independent item in the surgical skillset. For this reason, the visiting team was assembled with the purpose of providing training on all the essential components involved in a CABG operation. The “On-the-job” mentoring strategy, pairing up a team comprising each “player” in the surgical “orchestra”, turned out to be safe, efficient, and reproducible for key skill transfers. It resembled, to certain extent, the traditional surgical training of “see one, do one, teach one”. The fact that, in the last one of the three cases, the local team was able to perform the entire operation was a success from a technical point of view. Complete revascularizations were achieved in all cases, and there were no perioperative mortalities. These satisfactory results should be credited to meticulous case selection (with considerations of coronary targets, conduit conditions, heart function, patient age and risk profile, etc.) and surgical planning. One case was complicated by a major stroke, of which the causes could have been multifactorial, such as postoperative AF and possible pre-existing cerebral vascular disease. In fact, stroke was the top leading cause of death in women in PNG in 2021 according to World Health Organization data [8]. This case alerted the Team to further examine modifiable details in preoperative risk stratification and prophylaxis for AF, intraoperative hemodynamic management (including anesthesia, perfusion, and surgical handling) and postoperative intensive care, and AF management (including beta-blockade and early anticoagulation, should it occur)—particularly, algorithms in response to medical emergencies such as stroke activation. Local perioperative protocols need to be consolidated, tested, and repolished in future programs.

The small case number in this report has unavoidably resulted in great limitations for statistical analysis and the generalizability of the results. Nevertheless, this pilot CABG program serves as an important new page in the story of cardiac surgery in PNG. It serves not only as a landmark clinical victory in the establishing of a comprehensive national cardiac program but also as a significant milestone in the history of this country during its 50th year of independence. However, the journey ahead is challenging. Cardiovascular disease remains highly prevalent and largely untreated in LMICs, and at least 55.1 procedures per 100,000 people per year are estimated to be required to meet the population’s needs [9]. This is far beyond what is being met based on current capacity and capability. The training of a cardiac surgical team mandates long-term commitment as concerns time, manpower, and financial support. Achieving a certain cardiac surgery caseload depends on the complex function of infrastructural and workforce capacity, case detection via primary screening and referrals, and adequate health care coverage. Furthermore, the mere availability of a cardiac team does not necessarily translate to access to cardiac surgery. Adequate preoperative (e.g., cardiology, imaging, laboratory services), intraoperative (e.g., blood banking, cardiopulmonary bypass support), and postoperative services (e.g., intensive care, cardiology follow-up, and anticoagulation services) are essential to facilitate true access to timely, quality, and holistic cardiac surgical care in LMICs. International collaborations through the model of ‘‘twinning programs’’, like our case, by matching an evolving cardiac surgical center with an established cardiac center with experience, have proven to be effective and will continue to be effective and cost-efficient for knowledge transfer and capacity building [10,11]. Additional humanitarian efforts made by nongovernmental organizations to support such collaborations will also play a critical role in facilitating the local center’s growth [7].

In order to provide a sustainable cardiac surgical service in PNG, regular mission trips targeting a higher case load will be required, expectedly in the next five years, to further expand the capacity of the local team. Focus will be placed on improving the clinical experience in perioperative care across multiple disciplines. The introduction of disease-specific programs (such as ischemic heart disease and rheumatic valve disease) is also an aim, in order to refine the management of subgroups of patients and to form regional guidelines for them [12]. Since the launch of the CABG program in Aug 2025, one adult valve surgery program and one pediatric cardiac surgery program were also successfully completed in 2026. In the long term, strategic and specific collective commitment by all stakeholders and true collaborative partnership among mentee–mentor hospitals and international cardiac surgical societies will be keys towards the goal of establishing an independent, sustainable, and high-quality cardiac surgery program in PNG [13].

## 5. Conclusions

International collaborations, with the strategic planning of a “regionalization model”, can play a critical role in launching new cardiac surgical programs in LMICs, with acceptable surgical outcomes. History has been made with the first-ever successfully performed CABG operation, serving as a transformative moment in PNG’s medical care. The journey ahead is still long and challenging, and the aim is to strengthen and sustain local cardiac surgical capacity to ultimately provide safe, accessible, and affordable cardiac surgical care for the country.

## Figures and Tables

**Table 1:** Patients’ characteristics and perioperative variables.

Case No.	1	2	3
Age (years)	45	51	59
Gender	Male	Female	Male
Body mass index (kg/m^2^)	30.9	29.1	26.8
Diabetes mellitus	No	No	No
Hypertension	No	Yes	Yes
Dyslipidemia	Yes	Yes	Yes
eGFR (mL/min/1.73 m^2^)	115	79	140
LVEF (%)	46	66	48
NYHA	II	II	II
EuroSCORE II (%)	0.76	0.93	0.98
Angiogram	LM 50%, pLAD CTO, pLCx 70%, pRCA CTO	LM 70%, pLAD CTO, pLCx 80%	pLAD CTO, RCA 60%
No. of grafts	3	2	2
Grafts performed	LIMA-LAD, left radial-OM, SVG-RPDA	LIMA-LAD, SVG-OM	LIMA-LAD, SVG-RPDA
CPB (min)	101	68	107
AXC (min)	56	38	81
ICU stay (days)	1	1	1
Hospital stays (days)	15	34	16
30-day mortality	No	No	No
Morbidity	Nil	Stroke	Nil

LM: left main; pLAD: proximal left anterior descending; CTO: chronic total occlusion; pLCx: proximal left circumflex; pRCA: proximal right coronary artery; LIMA: left internal mammary artery; OM: obtuse marginal; SVG: saphenous vein graft; RPDA: right posterior descending artery; CPB: cardiopulmonary bypass; AXC: aortic cross-clamp.

## Data Availability

The original contributions presented in this study are included in the article material. Further inquiries can be directed to the corresponding author.

## Abbreviations

The following abbreviations are used in this manuscript:

PNG	Papua New Guinea
LMIC	low- and middle-income country
CABG	coronary artery bypass grafting
OT	operation theater
ICU	intensive care unit
LIMA	left internal mammary artery
CPB	cardiopulmonary bypass
AXC	aortic cross-clamp
AF	atrial fibrillation
POD	postoperative day
NYHA	New York Heart Association
